# Aquaporin Biology and Nervous System

**DOI:** 10.2174/157015910791233204

**Published:** 2010-06

**Authors:** Buffoli Barbara

**Affiliations:** Division of Human Anatomy, Department of Biomedical Sciences and Biotechnologies, University of Brescia, V.le Europa 11, 25123 Brescia, Italy

**Keywords:** Aquporins, nervous system, biology.

## Abstract

Our understanding of the movement of water through cell membranes has been greatly advanced by the discovery of a family of water-specific, membrane-channel proteins: the Aquaporins (AQPs). These proteins are present in organisms at all levels of life, and their unique permeability characteristics and distribution in numerous tissues indicate diverse roles in the regulation of water homeostasis.

Phenotype analysis of AQP knock-out mice has confirmed the predicted role of AQPs in osmotically driven transepithelial fluid transport, as occurs in the urinary concentrating mechanism and glandular fluid secretion. Regarding their expression in nervous system, there are evidences suggesting that AQPs are differentially expressed in the peripheral versus central nervous system and that channel-mediated water transport mechanisms may be involved in cerebrospinal fluid formation, neuronal signal transduction and information processing.

Moreover, a number of recent studies have revealed the importance of mammalian AQPs in both physiological and pathophysiological mechanisms and have suggested that pharmacological modulation of AQP expression and activity may provide new tools for the treatment of variety of human disorders in which water and small solute transport may be involved.

For all the AQPs, new contributions to physiological functions are likely to be discovered with ongoing work in this rapidly expanding field of research.

## 1. WATER IN LIVING CELLS

Water is the main component of all living cells, and water exchange may be considered as a property of life. At the extracellular level, water is the main component of biological fluids; at the extracellular/intracellular interface, water exchange through the plasma membrane maintains the osmolarity of the cytoplasm and thus the integrity of the cells. At the molecular level, water is involved in the configuration of some important molecules. Indeed, water is a polar molecule and displays electrostatic properties, forming hydrogen bonds, that confer its capacity of interacting with solutes but also participates in the stability and specificity of protein-protein and protein-DNA interaction. Water is also involved in metabolic processes such as cellular respiration and photosynthesis in plants.

The biological membrane surrounding living cells is not a pure lipid bilayer. There is a simple diffusion of water through a lipid membrane but it does not explain the high velocity of water exchange observed, for instance, in red blood cell membrane, nor the low energy activation observed for such phenomena [2]. Furthermore, first measurements of water movement and its effective re-absorption through the kidney proximal tubules comforted the hypothesis of some specific pathways for water molecules trafficking [19]. In accordance to previous data, these observations strengthened the idea of existence of selective water pores in biological membrane. Many clues of their presence were then provided in a broad variety of organs such as in salivary glands, eyes and brain [26].

## 2. DISCOVERY OF AQUAPORINS

The existence of water channel proteins was suspected long before their identification from experiments showing that red blood cells membranes are more permeable to water than expected from water diffusion through a lipid bilayer [87]. Further studies revealed that the water permeability of red blood cells could be inhibited by mercurial compounds (HgCl_2_) and that the inhibition could be reversed with chemical reducing agents. Taken together, these studies suggested that water transport occurs through a protein that has free sulfhydryl groups that are accessible to mercury [57].

The molecular basis for the water transport phenomenon was first identified by the team of Peter Agre. A 28 kDa protein, subsequently known as CHIP28 and re-named AQP1 for Aquaporin 1, was purified from red blood cells and renal proximal tubule membranes [3, 22] and then characterized as a water channel showing that *Xenopus Laevis* oocytes expressing AQP1 in their plasma membrane were far more susceptible to osmotic lysis than non-expressing oocytes [77].

The corresponding cDNA was cloned and the deduced aminoacid sequence showed high homology with the ancient family of membrane channels, MIP for Major Intrinsic Protein [76] and, in particular, with MIP of the eye lens [32], which was later named AQP0.

In parallel, studies on the antidiuretic hormone (ADH) responsive cells in amphibian urinary bladder led to the idea that changes in water permeability in ADH-sensitive cells result from the insertion in apical plasma membrane of new components that contains channels for water [35] and so to the discovery of the second water channel protein, AQP2 [68, 69]. Since 1993 several AQPs have been discovered in organisms for all kingdoms of life [85], including unicellular organisms (bacteria, yeast and protozoa) and multicellular ones (plants, animals, and humans) underlying the importance of these channels for life [46].

A subsequent functional characterization has given new insights regarding the role of these proteins that for some members is not restricted or not linked to water movement but to the transport of non-ionic and/or small neutral solutes.

## 3. THE AQUAPORIN FAMILY

The water channel family is still growing with 13 members ubiquitously distributed in mammalian tissues [47, 94]. Eleven of the 13 members have been identified from various mammalian tissue including the nervous system, kidney, gastrointestinal tract, skin and respiratory tract [52, 53].

These channels have been highly conserved throughout evolution and the family is now divided accordingly to sequence homology and permeability [5] into: *aquaporins* and *aquaglyceroporins*.

The subgroup of *aquaporins* is composed of AQP0, AQP1, AQP2, AQP4, AQP5, AQP6 and AQP8 and is considered to be mainly permeable to water with a high flow rate. A few of these pure water channels are also permeable to anion (AQP6), volatile solutes such as CO_2_ for AQP1 [20] and ammonia for AQP8 [43]. Water diffusion through AQPs is inhibited by mercury, except AQP4 which is a mercury-insensitive AQP [5].

The second subgroup *aquaglyceroporins*, is composed of AQP3, AQP7, AQP9, AQP10 and bacterial glycerol facilitator (Glpf) [10]. These channels are permeable to water, glycerol and urea. AQP9, a member of this group, was also surnamed “neutral channel” [92]. Indeed, the presence of AQP9 in *Xenopus Oocytes *or proteoliposomes injected facilitated the diffusion of water, but also polyols (glycerol, mannitol, and sorbitol), purines (adenine), pyrimidines (uracil and chemotherapeutic agent 5-fluorouracil) and monocarboxylates (lactate, β-hydroxybutyrate) [18, 41, 48, 92]. However, the osmotic water coefficient for AQP9 is lower than in a pure water channel like AQP4 [18]. In addition AQP9 facilitates metalloid transport further suggesting that AQP9 may be a major route of arsenite uptake into mammalians cells [54].

In addition to aquaporins and aquaglyceroporins, a third subfamily of related proteins was discovered later by Ishibashi [40], being called “*superaquaporins*” or “*subcellular aquaporins*”. Originally, in this subfamily were included the mammalians AQP11 and 12; recently, the subfamily was renamed as “*unorthodox AQPs*” and mammalian AQP6 and 8 were also included. Few papers have been published on them and this may be because their functions have not been clearly shown and they seem to be localized inside the cells [71].

## 4. MOLECULAR STRUCTURE OF THE AQUAPORINS

The configuration of AQP1 reflects the common structural features of the AQP family with aminoacid similarity from 20% to 50%. The primary sequence of the cDNA revealed that AQP protein monomer has a molecular weigh of around 30 kDa and subunits comprise six alpha-helix transmembrane domains with an inverted symmetry between the first and last three domains [76]. The two connecting loops, between transmembrane helices 2-3 and 5-6 contain an aminoacid triplet with the sequence Asn-Pro-Ala (NPA) conserved across members of the AQP family and implied in pore formation and water selectivity Fig. (**1A**) [1, 9].

Biochemical and freeze fracture studies have indicated that, as with other channel like membrane proteins, functional AQPs assemble in cell membranes as tetramers with intracellular N- and C-termini [1, 95]. However, in contrast to most ion channel, the permeation pathway does not reside at the axis of symmetry formed by the four constituent subunits, but rather each subunit monomer contains separate pores [93]. Thus, each fully assembled AQP contains four channels for water permeation Fig. (**1B**).

The pore narrows midway between the leaflets of the bilayer to approximately between 3 and 6 Å diameter [37]. The specificity for water seems to be due to the diameter of the pore [33]. Moreover, because under different physiologic stimuli, water may be taken up or released by cells, the molecular architecture for a water-selective transporter must explain bi-directionality.

The molecular basis for AQP substrate specificity has been also widely investigated and it was demonstrated that, in spite of a general highly conserved structure, differences at the key area of the AQP sequence are responsible for the differences in channel selectivity [28, 51]. This variability at the primary structure defines two constriction points within the pore, referred to as the NPA constriction and the aromatic/arginine selectivity filter, respectively and the selection is done through mechanisms of charge, polar and size exclusions [38].

## 5. REGULATION OF AQUAPORIN PERMEATION

Although the movement of water through AQPs is primarily driven by osmotic gradients, there is evidence that permeation can be modulated by external factors.

AQP3 permeability is reduced at acidic pH, whereas the water and ion permeability of AQP6 is enhanced under similar condition [39, 102, 103]. Furthermore, it has been reported that the water permeability of AQP4 is reduced following protein kinase C activation [34] and that AQP1 could be gated by cyclic GMP [6, 13].

It has long been known that water transport in many cells is sensitive to inhibition by mercury-containing compounds, and the permeability of many human AQPs is also affected by these substances. Early studies demonstrated that AQP1 has a cysteine residue (C189) near the NPA motif and its mutation to serine results in a loss of inhibition by mercury [78]. Similar pore loop cysteine residues are present in other mercury-sensitive AQPs [50]. By contrast, the mercury insensitive aquaporin, AQP4, lacks cysteines at any of known mercury-sensitive sites [44].

Because of its toxicity and other pharmacological actions, mercury is not the ideal inhibitor to characterize AQP function. Unfortunately, there are only few other agents that inhibits AQPs: silver salts inhibit AQP1 [70]; tetraethyl ammonium, which is widely used as a blocker of potassium channels, blocks AQP1 [16]; and phloretin is a relatively potent inhibitor of several AQPs, including AQP9 [92]. However, phloretin lacks specificity, exhibiting inhibitory activity against solute transporters, as well as channels [24].

The absence of good pharmacological modulators has presented a challenge for defining the function of AQPs in physiological and pathophysiological processes. Indeed, much of what is known about AQP function has been determined using non-pharmacological strategies. mRNA and protein expression studies have helped to define potential roles by characterizing tissue, cellular and subcellular AQP localization. The association of genes encoding AQPs with human hereditary disorders has also aided understanding of AQP function. Some of the most significant advances in defining physiological functions for water-conducting channel have come from the detailed phenotype analysis of transgenic mice lacking genes encoding AQPs.

## 6. LOCALIZATION AND FUNCTIONAL ROLES OF AQUAPORIN IN MAMMALS

AQPs are relatively ubiquitous in mammalian organism and are usually not restricted to a sole tissue [95]. However, their specific distribution in certain cell types of an organ often reflects a precise function. Because they increase water membrane permeability, AQPs are important actors of fluid homeostasis maintenance and secretion/re-absorption.

AQP0 is present in the eye lens and its mutation provokes cataracts formation [94]. AQP1, the first characterized AQP, is mostly expressed in red blood cells, kidneys, peripheral and central nervous system (CNS), lungs and is associated with water re-absorption and fluid secretion [14]. AQP1 knock-out mice and humans with AQP1 mutation have shown reduced urinary concentration [45, 56]. AQP2 is permeable to water and is expressed in kidney collecting duct [95]; its mutations can cause an autosomal form of hereditary nephrogenic diabetes insipidus in humans [21]. In addition, recent data reported the expression of AQP2 isoform also in the nervous system [15, 17]. AQP3 is also expressed in different tissues including kidney collecting duct, skin, conjunctiva of the eye, oesophagus, colon, spleen, stomach, small intestine, urinary bladder and respiratory tract airway epithelium. It is permeable to water and glycerol and may be important for proper skin hydration and elasticity [81, 95]. Mice lacking AQP3 manifest nephrogenic diabetes insipidus with polyuria, polydipsia, and urinary hypoosmolality [96]. AQP4 is permeable to water and expressed in astroglial cells at blood–brain barrier and spinal cord, kidney collecting duct, glandular epithelia, airways, skeletal muscle, stomach and retina [95]. In the brain, AQP4 null mice showed altered cerebral water balance with protection from brain edema [58]. AQP5 is permeable to water and expressed in glandular epithelia, corneal epithelium, alveolar epithelium and gastrointestinal tract [95]. Saliva production in the parotid gland is a sympathetically controlled phenomenon which involves the re-localization of AQP5 into the plasma membrane of the secretory cells [42]. The important role of AQP5 in homeostasis is evidenced by AQP5 knock-out mice which have reduced fluid secretions [95]. Defective cellular trafficking of AQP5 in salivary [89] and lacrimal [91] glands has been associated to the Sjörgren's syndrome, an autoimmune disease characterized by deficient secretion of tears and saliva. AQP6 is thought to be involved in chloride permeability and is expressed in kidney collecting duct intercalated cells [95]. AQP7 is involved in water and glycerol permeability and is expressed mainly in adipose tissue, and in testis, heart, skeletal muscle and kidney proximal tubule [95]. In adipocytes, AQP7 is known to facilitate the secretion of glycerol, which constitutes a key metabolite in the control of fat accumulation and glucose metabolism [80]. AQP8 is permeable to water and is expressed in liver, pancreas intestine, salivary gland, testis and heart [95]. It was suggested to be involved in cAMP stimulated bile secretion by Garcia and colleagues [30]. AQP9 is expressed in liver, white blood cells, testis and brain, and is involved in water and small solutes permeability [95]. The functional role of AQP9 in the brain is still unknown, but its permeability to glycerol and lactate indicates that it may play a role in energy metabolism [5, 90]. Studies performed with mice lacking AQP9 and leptin receptor function, developed obese mice, with type II diabetes and increased lipolysis and glycerol production [82]. AQP10 is thought to be involved in water and glycerol permeability and is expressed in small intestine [95]. AQP11 is expressed in kidney, testis, liver, brain, intestine, heart, and adipose tissue, but its function is still unknown [31, 81, 95]. AQP12 seems to be expressed specifically in pancreatic acinar cells, and as well as AQP0 and AQP11, it has not been characterized functionally. AQP12 knock-out mice have not been generated, and no human diseases have been credited to mutations in the AQP12 gene [81, 95]. The involvement of AQPs in many human diseases such as glaucoma, obesity or cancer, is now well reported. Indeed, AQPs are strongly expressed in tumour cells and promote cell migration [84]. Migration is a fundamental property of cells that occurs during many physiological and pathological processes including organogenesis in the embryo, repair of damaged tissue after injury, the inflammatory response, formation of new blood vessels, and the spread of cancer. AQP dependent cell migration has been found in a variety of cell types in vitro and in mice in vivo [75]. AQP1 deletion reduces endothelial cell migration, limiting tumour angiogenesis and growth. AQP4 deletion slows the migration of reactive astrocytes, impairing glial scarring after brain stab injury. AQPs are conducive to migration by facilitating the rapid cell volume changes and augmenting cell propulsion. The emerging roles of water movement in cell migration are not only important in the mechanistic understanding of the migration process, but may also have a wide range of therapeutic implications [75]. Thus, the modification of AQP function or expression constitutes a promising target to develop new drugs and provide a treatment of several diseases [97].

## 7. AQUAPORINS IN THE NERVOUS SYSTEM

Several recent studies have shown that AQPs are important in the nervous system homeostasis and neuronal signaling. The AQP family in the CNS has diverse functions including that of a bidirectional water pathway between the brain and blood vessels, cerebrospinal fluid formation (CSF), neural signal transduction, osmoreception and some pathophysiological conditions [52]. To date, only some AQP subtypes (AQP1, 3, 4, 5, 8, 9) have been reported in the CNS being identified in choroidal cells (AQP1), astrocytes (AQP1, 3, 4, 5, 8, 9), oligodendrocytes (AQP8), neurons (AQP1, 5, 8), tanycytes (AQP9) and ependymal cells (AQP1, 4, 9) [8, 52, 59, 60, 67]. 

In contrast to numerous studies of AQP localization and function in the CNS, little information is available on the expression and function of AQPs in peripheral nervous system (PNS).

### 7.1. AQP1

In rodents AQP1 is found on epithelial cells of choroids plexus, where it was concentrated at the apical pole [59, 68] and plays a role in CSF [73]. AQP1 is up-regulated in choroids plexus tumours [55], which are associated with increased CSF production, against supporting a role for this isoform in CSF secretion. AQP1 is not found in normal brain capillary endothelium, although highly expressed in peripheral endothelial cells [23, 68]. Cerebral capillary endothelial cells cultured in the absence of astrocytes [23] and capillary endothelial cells within the brain tumours (which are not surrounded by astrocyte end-feet) express AQP1 [83]. These observations suggest that astrocyte end-feet may signal adjacent endothelial cells to switch of endothelial AQP1 expression.

Moreover, recently a novel role of AQP1 in spinal cord injury was reported. In particular, they reported that constant hypoxic conditions in chronically injured spinal cords persistently elevate AQP1 in neurons, ependymal cells, astrocytes, and sensory fibers and so may contribute to different pathological condition after spinal cord injury, such as neuronal/axonal swelling, over-production of CSF and formation of cysts, or excessive axonal sprouting underlying the development of neuropathic pain and autonomic dysreflexia [64]. 

Regarding its distribution in PNS AQP1 is found in small diameter sensory neurons in dorsal root, trigeminal and nodose ganglia, but studies of a possible role for AQP1 in pain sensation remains to be determined [74, 86]. Moreover, expression of AQP1 protein was found in glial cells of the PNS where have extensive roles in regulating extracellular concentration of ions, metabolites and neurotransmitters [88]. Increasing evidence indicates that glial cells function to coordinate the differentiation, metabolism and excitability of neurons, to modulate synaptic transmission and to integrate signals emanating from neurons and other glial cells [7, 25]. In addition, AQP1 expression in these may provide a membrane marker to distinguish these cells from glial cells of the CNS that express AQP4 and AQP9 [29].

Other recent studies have demonstrated the presence of neuronal elements for AQP1 in the enteric nervous system [62] and in the mechanoreceptive Ruffini endings in the periodontal ligament [63].

### 7.2. AQP4

AQP4 is the principal isoform in mammalian brain. It was first cloned from rat lung tissue [36] and shown to be expressed strongly in brain [101]. AQP4 is expressed in astrocytes and ependymal cells throughout the brain and spinal cord, particularly at the sites of fluid transport at the pial and ependymal surfaces in contact with the CSF in the subarachnoid space and the ventricular system [67, 79]. Polarized AQP4 expression is found in astrocytic foot process in direct contact with blood vessels. In spinal cord, AQP4 expression is high in gray matter where numerous AQP4 dense process are found in direct contact with neuronal cell bodies and synapses [72, 99]. Verkman and co-workers [98] reported three distinct roles of AQP4 in brain function by phenotype analysis of AQP4 knock-out mice: the involvement in brain edema, in glial cell migration and in neuronal sign transduction. In particular, AQP4 facilitates clinically important water movement into and out of the brain in the development and resolution of brain edema and modulation of AQP4 expression or function is also predicted to modulate glial scar formation, which may be of clinical utility in traumatic injury, tumour and infection; moreover, recent data suggest increase extracellular space volume in AQP4 deficiency and impaired K^+^ reuptake by AQP4-null astrocytes, which may be related to functional significant AQP4-K^+^ channel interactions [12]. 

### 7.3. AQP9

AQP9 is a subtype mainly expressed in liver and testis having characteristics of being permeable to various solutes, including glycerol and lactate and this subtype might have a role in energy metabolism, as demonstrated by its presence in mitochondrial inner membranes [4]. In the brain, this water and solute channel is present in the cells surrounding the cerebral ventricules, including ependymal cells and the tanycytes [10], and is expressed in astrocytes, brain stem catecholaminergic neurons [9] and subsets of midbrain dopaminergic and hypothalamic neurons [4]. A recently evidence of the presence of tetrameric AQP9 in brain and of expression in neurons has been provided by the analysis of mice with targeted deletion of AQP9 gene [61].

In general, AQP9 expression becomes up-regulated in several brain diseases [10], for example in astrocytes bordering cerebral infarcts in mice [90]. Moreover, similarity of distribution pattern within the brain between AQP4 and AQP9 in mice and rats suggest that the two proteins mediate common function, and can act in synergy contributing to the facilitation of water movements between CSF and brain parenchyma [10].

### 7.4. Other Aquaporins

Reverse transcription-polymerase chain reaction showed expression of AQP3, AQP5 and AQP8 in neuronal primary cultures and astrocyte cultures. Whereas microglial cells did not express AQP in rat brain, AQP8 was observed in oligodendrocytes and AQP5 in astrocytes [100]. Moreover AQP8 was detected primarily in ependymal cells lining the fluid-filled central canal of mouse spinal cord [72]. Nevertheless, the physiological role of this protein remains to be determined.

A new role of AQP2 isoform in PNS has recently been established. The data showed, for the first time, the expression of this protein in trigeminal and dorsal root ganglia in response to pain stimuli and in particular, respectively, inflammatory and neuropathic pain [15, 17].

## 8. AQUAPORINS: A PROMISING TARGET FOR DRUG DEVELOPMENT

Water transport is a fundamental process contributing to human physiology and pathophysiology. Therefore, target pharmacological modulation of water and solute transport using AQPs would appear to provide novel opportunities for therapeutic treatments in a variety of human diseases, such as brain edema, glaucoma, tumour growth, congestive heart failure and obesity.

To date, there are few pharmacological modulators of AQPs available, and those that are known lack specificity or are toxic. Furthermore, the broad tissue expression of AQP subtypes in human will probably necessitate the identification and development of subtype-selective AQP modulators.

Discovering new AQP modulators presents a challenge because screening strategies commonly used in the pharmaceutical industry to identify modulators of ion channels, G-protein-coupled receptors and enzymes might not be appropriate for water channels. Moreover, there are not known specific high-affinity ligands for AQPs that can be used in radioligand binding assays. 

To date, there are increasing of evidence that AQPs allow the passage of metalloid compounds; in fact, trivalent arsenic an antimony compounds, at physiological pH, behave as molecular mimics of glycerol and are conducted through AQP channels. Despite their toxicity, both metalloids are used as chemotherapeutic agents for the treatment of cancer, so the understanding of the factors that modulate AQP expression will provide a new approach to metalloid based chemotherapy [11].

Moreover, recent data suggest the potential use of some recent therapeutic approaches, such as RNA interference (RNAi) and immunotherapy [27].

An intriguing issue is how the expression of AQPs is modified by drugs used for the treatment of brain edema caused by trauma, tumour and cerebral haemorrhage, but its mechanism of action is not known [49]. In a recent study, RNAi technology has been used to specifically suppress AQP4 expression in astrocytes primary cultures and so to elucidate its functional role in glial cells [65, 66]. Although it is unlikely that RNAi could be used as an efficient drug to rapidly block astrocytes swelling, AQP4 inhibition studies by RNAi in animals will be very useful to test the hypothesis that the inhibition of AQP4 activity is a potential target for the treatment of brain edema. Moreover, RNAi could be used to inhibit other AQPs expression in those pathologies where long term overexpression occurs, such as in tumour angiogenesis.

To date, it is still necessary to develop specific strategies for the discovery of potent pharmacological modulators that may be used as reversible AQP blockers and agents that target AQPs in specific tissues.

## CONCLUSION

Future research should clarify the physiological roles of AQPs in different tissues and cells where the role is not yet obvious. An important direction of study is represented by evaluation of new methods for diagnosis and therapy diseases in veterinary and human medicine based on progress in understanding the precise cellular localization and the regulation of the expression of these water channels in various cells.

## Figures and Tables

**Fig. (1):**
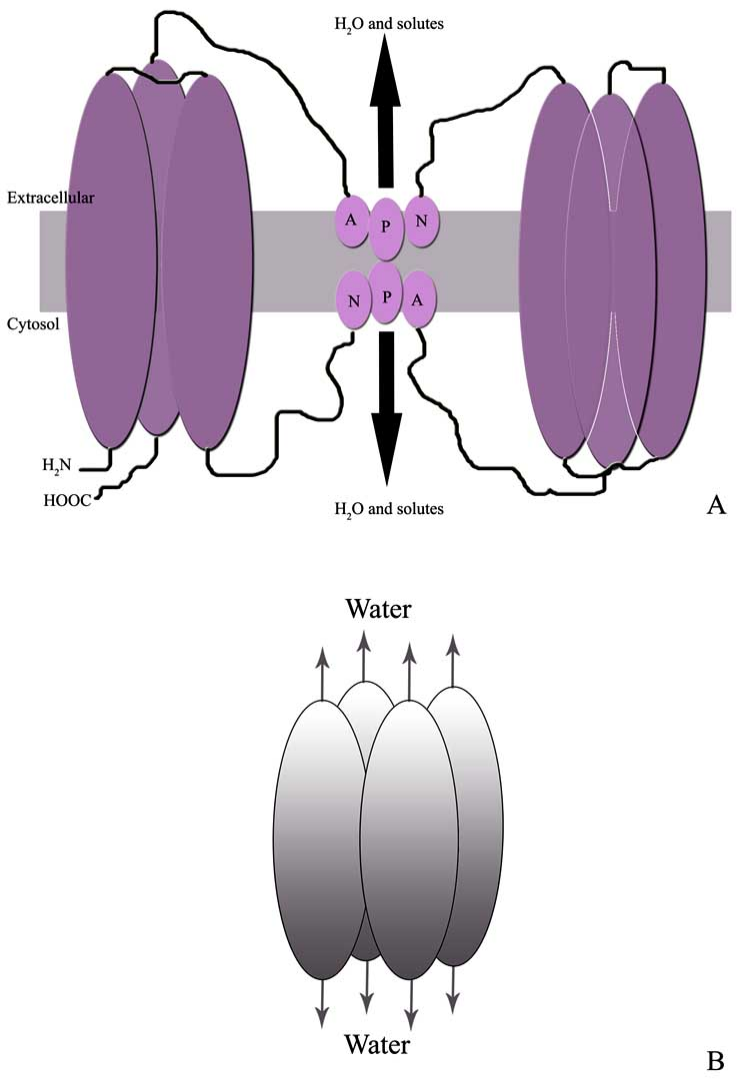
Schematic representation of the common AQP structure (**A**); tetrameric arrangement of AQP membrane. Each monomer has a water pore (**B**).
