# ERRATUM

**DOI:** 10.2174/157015912800604506

**Published:** 2012-06

Due to an overlook on the author's side, author’s name in the article entitled as “**Aquaporin biology and nervous system**” by, **Buffoli B** published in the journal “*Current Neuropharmacology*” Vol. 8, Issue no. 2, was wrong. The correct name is as follows:

Buffoli B. Aquaporin biology and nervous system. Curr Neuropharmacol 2010, 8(2), 97-104. 97-104

